# First Report of *Orthonairovirus songlingense* in *Haemaphysalis concinna* Ticks from Russia

**DOI:** 10.3390/v18060688

**Published:** 2026-06-22

**Authors:** Mikhail Y. Kartashov, Valentina Y. Kurushina, Kirill A. Svirin, Alina S. Zheleznova, Tatyana V. Tregubchak, Alexander P. Agafonov, Anastasia V. Gladysheva

**Affiliations:** State Research Center of Virology and Biotechnology “Vector”, 630559 Kol’tsovo, Russia; kartashov_myu@vector.nsc.ru (M.Y.K.); kurushina_vyu@vector.nsc.ru (V.Y.K.); svirin_ka@vector.nsc.ru (K.A.S.); zheleznova_as@vector.nsc.ru (A.S.Z.); tregubchak_tv@vector.nsc.ru (T.V.T.); agafonov@vector.nsc.ru (A.P.A.)

**Keywords:** ixodid ticks, *Haemaphysalis concinna*, *Orthonairovirus*, emerging virus, Songling virus, identification, sequencing, phylogenetic analysis

## Abstract

High-throughput sequencing methods have made it possible to identify numerous novel tick-borne viruses that are potentially pathogenic to humans. Among these, Songling virus (*Orthonairovirus songlingense*, SGLV) has been associated with febrile illness in patients following tick bites in China, but its geographic distribution outside China remains largely unexplored. In this study, we aimed to detect SGLV circulation in ticks across Asian Russia, focusing on regions bordering China. A total of 3444 adult ticks representing six species were collected from 170 locations across 11 regions during the summer of 2024. SGLV RNA was detected in *Haemaphysalis concinna* ticks, with 11 positive specimens yielding an SGLV RNA prevalence rate of 2.2%. Positive ticks were found in four regions, with the highest positivity rate (5.8%) recorded in Amur Oblast, which directly borders China. The detection of SGLV in the Republic of Altai represents the westernmost record of this virus to date. Full-length nucleoprotein-coding sequences obtained for all Russian isolates revealed up to 1.2% nucleotide divergence. Phylogenetic analysis confirmed that all Russian SGLV isolates belong to *Orthonairovirus songlingense*, with the Altai SGLV isolate showing genetic similarity to a human-derived Chinese SGLV isolate. Co-infections with *Rickettsia heilongjiangensis* were detected in four SGLV-positive ticks, highlighting the potential for simultaneous pathogen transmission. These findings establish the first evidence of SGLV circulation in Russia across a wide geographic range and underscore the need for differential diagnosis of febrile illnesses following tick bites in this region.

## 1. Introduction

Arboviruses (arthropod-borne viruses) constitute a large, taxonomically heterogeneous group of viruses united by a common ecological characteristic. Their transmission via the bites of blood-feeding arthropods, primarily mosquitoes and ticks. Within the arthropod vector, arboviruses establish a persistent infection, replicating primarily in the epithelial cells of the midgut and salivary glands without causing overt pathology. This ensures efficient transmission of the pathogen during subsequent blood feeding. A defining feature of arboviruses is their remarkable ability to alternate between invertebrate vectors and vertebrate hosts, known as the vector-host switch [1].

Ixodid ticks transmit a wide range of arboviruses pathogenic to humans and animals. A significant number of tick-borne viruses capable of causing diseases in humans and mammals with a wide range of clinical manifestations belong to the *Orthonairovirus* genus of the Nairoviridae family. Crimean-Congo hemorrhagic fever virus (*Orthonairovirus haemorrhagiae*, CCHFV) has the greatest medical significance. It is endemic across Africa, Asia, the Middle East, and parts of Europe, including southern Russia. CCHFV causes severe hemorrhagic fever with a 10–40% fatality rate, lacks licensed vaccines or specific antivirals, and is prioritized by WHO for epidemic preparedness [2,3]. The genome of orthonairoviruses consists of a single-stranded negative-sense RNA (ssRNA(-)) and is divided into three segments: L, M, and S. Currently, the *Orthonairovirus* genus comprises 52 species and a number of unclassified viruses [4].

Metagenomic approaches have enabled the identification of numerous novel tick-borne viruses pathogenic to humans, including within the *Orthonairovirus* genus. Among the recently discovered tick-borne orthonairoviruses is Songling virus (*Orthonairovirus songlingense*, SGLV). SGLV is a novel tick-borne orthonairovirus associated with febrile illness in humans that was first identified in both humans and ticks in Northeast China [5].

SGLV has a genomic organization typical of the *Orthonairovirus* genus. The L segment is approximately 12 kb in length and encodes the large (L) protein, which functions as the RNA-dependent RNA polymerase and consists of 3951 amino acids. The M segment is approximately 4 kb long and encodes a 1355-amino acid polyprotein that is processed into the viral glycoproteins. The S segment is approximately 1.5 kb in length and encodes the 487-amino acid viral nucleoprotein. Sequencing the S segment is of particular importance not only for broad phylogenetic surveillance but also because the nucleoprotein plays a central role in viral RNA encapsidation and ribonucleoprotein (RNP) assembly. Our recent structural studies have revealed critical features of the SGLV nucleoprotein, including a highly conserved positively charged RNA-binding crevice and specific RNP conformational dynamics, despite low sequence identity with other orthonairoviruses. Therefore, obtaining new S segment sequences allows for mapping newly identified amino acid substitutions onto these structural models, providing deeper insights into its structural plasticity [6].

SGLV strains isolated from patients demonstrated the ability to induce pronounced cytopathic effects in the SMMC-7721 cell line. Recently studies have demonstrated the pathogenic impact of SGLV on human health, manifesting as symptoms such as fever, general asthenia, headache, and dizziness [5]. SGLV was detected in 42 out of 658 hospitalized patients who had suffered tick bites in China [5]. Serological studies revealed the presence of virus-specific antibodies in 69% of patients during the acute phase of the disease. Notably, SGLV infection typically presents with nonspecific symptoms that overlap with other tick-borne pathogens, both viral (e.g., Yezo virus, Tacheng tick virus 1) and bacterial (e.g., *Rickettsia sibirica*) [7,8,9,10,11]. This significantly complicates differential diagnosis based on clinical symptom analysis, suggesting that the actual contribution of this virus to infectious pathology may be significantly greater than currently recognized. SGLV genetic material has been detected in tick species such as *Ixodes crenulatus*, *Haemaphysalis longicornis*, *Haemaphysalis concinna*, *Haemaphysalis flava*, and *Ixodes persulcatus* in China [12,13,14]. Investigations into potential warm-blooded hosts have demonstrated the presence of viral RNA in 2.2% of spleen samples from great gerbils (*Rhombomys opimus*) [15].

The geographic proximity of the vast territories of Siberia and the Russian Far East to regions of China, where recent tick virome studies have revealed a significant diversity of pathogenic viruses, including SGLV, underscores the necessity of monitoring the full spectrum of known tick-borne pathogens in these areas [13,16]. This need is further compounded by the wide distribution of arthropod vectors and the continuous expansion of areas subject to anthropogenic impact.

The aim of this study was to detect the circulation of SGLV in ticks within Russia, focusing primarily on regions bordering China. The molecular analysis relied on sequencing the SGLV S segment, selected as the most conserved region of the orthonairovirus genome. This strategy was employed to ensure comprehensive detection of SGLV isolates circulating across the Russia, prioritizing broad surveillance over the assessment of genetic diversity. Establishing the presence and distribution of SGLV through this broad screening strategy is essential for accurate risk assessment and the timely implementation of preventive measures.

## 2. Materials and Methods

### 2.1. Tick Sampling and Collection

Tick collection was conducted by flagging vegetation across 170 locations within 11 administrative regions in Asian Russia. Sampling took place during the summer season of 2024 to coincide with peak tick activity. Collected specimens were morphologically identified to the species level using established taxonomic keys and subsequently stored at −80 °C for further study.

### 2.2. Generation of Tick Sample Collection Map

A custom Python script was developed to generate a map based on tick collection points. Analyses were performed using Python v3.12.7 with the following libraries: Plotly v6.0.0 for interactive visualization, Shapely v2.1.2 for determining the spatial relationship between points and administrative regions, Openpyxl v3.1.5 for handling source data in Excel format, Requests v2.32.5 for retrieving GeoJSON data, and NumPy v2.4.1 and Pandas v2.3.3 for data manipulation and analysis.

### 2.3. Nucleic Acid Preparation

Prior to nucleic acid extraction, individual ticks were surface-sterilized by washing with 70% ethanol, followed by air-drying under sterile conditions to minimize external contamination. Subsequently, each tick was mechanically homogenized in 300 µL of sterile phosphate-buffered saline using a TissueLyser II homogenizer (Qiagen, Hilden, Germany) and metallic beads. The homogenates were then centrifuged at 10,000 rpm for 15 min (+4 °C) and the resultant supernatants were passed through sterile 0.22 μm filters. Total nucleic acids were extracted using the phenol-chloroform method with the LIRA+ reagent kit (Biolabmix, Novosibirsk, Russia). The extracted nucleic acids were eluted in RNase-free water and stored at −80 °C. Aliquots were used for nucleic acid quantification and complementary DNA (cDNA) synthesis. First-strand cDNA synthesis was performed via reverse transcription using MMLV reverse transcriptase and random decanucleotide primers (Evrogen, Moscow, Russia).

### 2.4. PCR Screening

Detection of SGLV RNA in the obtained samples was performed by quantitative PCR (qPCR) using a specific primer pair (5′-CAGCTTCCTGATACTGAAGGAC-3′ and 5′-GAGAAGAACAAGGACCACAAGA-3′) and a fluorescently labeled probe (HEX-TTTCAGGCTTTCGTACTCCTTGGACC-BQ1), which are complementary to a conserved fragment of the viral S segment [17]. The 25 µL reaction mixture was prepared using the BioMaster HS-qPCR (2×) reagent kit (Biolabmix, Novosibirsk, Russia). Amplification and fluorescence detection were carried out on a CFX96 Touch thermocycler (Bio-Rad, Hercules, CA, USA) under the following cycling conditions: polymerase activation at 94 °C for 5 min (1 cycle), followed by 45 amplification cycles consisting of denaturation at 94 °C for 10 s, primer annealing at 55 °C for 20 s, and extension at 70 °C for 20 s. Fluorescence signals in the HEX channel were acquired during the annealing step (55 °C, 20 s). Diethyl pyrocarbonate-treated water replaced cDNA in the negative control. A Ct value < 35 was considered positive. DNA detection of *Rickettsiae* spotted fever group was carried out using a pair of primers complementary to a fragment of the citrate synthase gene (gltA) [18]. Tick specimens were additionally tested for the presence of tick-borne encephalitis virus, *Borrelia burgdorferi* s.l., *Borrelia miyamotoi*, and *Anaplasma phagocytophilum* using previously described protocols [19].

### 2.5. Next-Generation Sequencing

To enable sequencing of the full-length protein-coding sequence of the S segment from detected SGLV genetic variants, cDNA was pre-enriched by multiplex PCR using a custom-designed primer panel (Table 1). This panel was designed to amplify overlapping fragments averaging ~600 nt in length. Adjacent fragments share an overlap region of ~150 nt. Primer design was performed using PerlPrimer v1.1.21 based on consensus sequences derived from multiple sequence alignment of available at the GenBank full-length SGLV S segment sequences [20]. Optimal oligonucleotide annealing temperatures and the likelihood of non-specific primer-primer interactions were evaluated using OligoCalc v3.19 (https://oligocalc.eu/; accessed on 21 May 2026).

Multiplex enrichment was performed in two separate reaction mixtures using an PCR amplification kit with Fusion 2.0 DNA polymerase (Biolabmix, Novosibirsk, Russia). Enrichment efficiency was assessed by 2% agarose gel electrophoresis, and double-stranded DNA concentration was quantified using a Qubit 2.0 fluorometer with the Qubit double-stranded DNA (dsDNA) HS Assay Kit (Thermo Fisher Scientific, Eugene, OR, USA). The resulting dsDNA fragments were purified from unspent components and reaction products with AMPure beads (Beckman Coulter, Brea, CA, USA), and then used to prepare NGS libraries.

Library preparation for high-throughput sequencing was performed using the NEBNext Ultra II FS DNA Library Prep Kit for Illumina (New England Biolabs, Ipswich, MA, USA). The workflow included DNA fragmentation, end repair, dA-tailing (adenine addition to 3′ ends), and adapter ligation using a single enzymatic mix. Sequencing was carried out on the Illumina MiSeq platform (Illumina, San Diego, CA, USA).

Primary processing of FASTQ files was performed using fastp v0.20.1 (https://github.com/OpenGene/fastp/; accessed on 21 May 2026) to remove adapter sequences and to filter reads by length (<30 nt) and quality (Phred score < 20). Preprocessed reads were aligned to the reference SGLV S segment sequence (GenBank ID: MT328777) using BWA-MEM v. 0.7.18 (https://github.com/lh3/bwa; accessed on 21 May 2026). Processing and analysis of aligned data in SAM/BAM formats were performed using Samtools v1.11. Consensus sequences were extracted from BAM files using iVar v1.2.2.

### 2.6. Phylogenetic and Sequences Analysis

All available complete protein-coding sequences were downloaded for SGLV (*n* = 15) from NCBI (https://www.ncbi.nlm.nih.gov/labs/virus; accessed on 24 March 2026) and their sequence information was recorded (Table S1). Multiple sequence alignment was performed using MEGA 11 software (PSU, Philadelphia, PA, USA) [21]. These programs were also used to calculate sequence identities. A custom Python script was developed using the Biopython library to compute pairwise sequence identity matrix. The analysis was performed using Python v3.12.7 with the following libraries: Biopython v1.85 for sequence parsing and manipulation, Pandas v2.2.3 for data handling, Seaborn v0.12.2, and Matplotlib v3.7.2 for visualization.

Phylogenetic trees were constructed using MEGA 11 software using the maximum likelihood method. Prior to tree inference, the optimal nucleotide substitution model was selected based on information criteria. Statistical support for tree topology was assessed by bootstrap analysis with 1000 pseudoreplicates. The resulting phylogenetic tree was visualized using iTOL v7.1 (https://itol.embl.de/; accessed on 24 March 2026) [22].

### 2.7. Nucleotide Sequence Accession Numbers

Nucleotide sequences determined in the study (*n* = 11 for SGLV and *n* = 2 for *Rickettsia*) are available in GenBank under accession numbers PX461779-PX461789, PX935561, and PX935562.

### 2.8. Statistical Analysis

The prevalence of SGLV RNA was estimated using 95% confidence intervals (95% CIs). The 95% confidence interval was calculated using Wilson’s estimator without correction for continuity (https://pedro.org.au/wp-content/uploads/CIcalculator.xls; accessed on 21 May 2026).

## 3. Results

### 3.1. Tick Sample Collection

**Figure 1:**
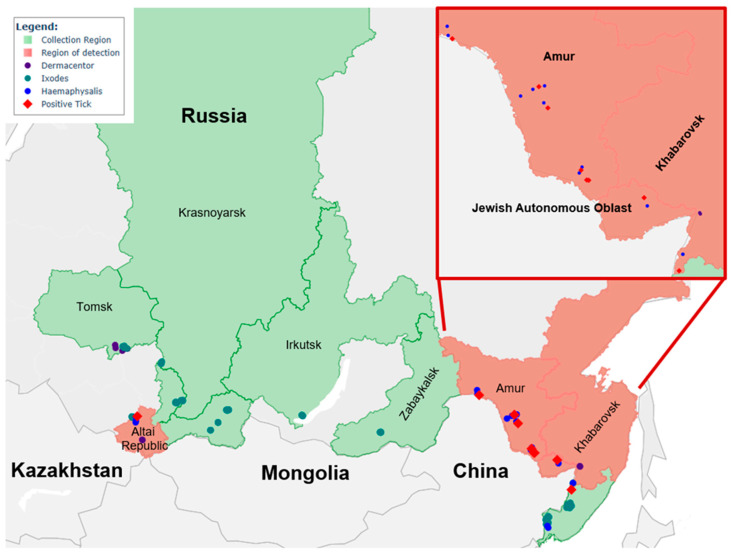
Geographic map of sample collection sites in Russia. Regions where ticks were collected are highlighted in green to clearly indicate the administrative affiliation of collection points, particularly for sites located near regional borders. Regions with SGLV RNA-positive ticks are highlighted in red. Collection sites for ticks of the *Dermacentor* spp. are marked with purple circles, *Ixodes* spp. with green circles, and *Haemaphysalis* spp. with blue circles. Locations where SGLV-positive ticks were found are marked with a red rhombus.

A total of 3444 adult ticks were collected across 11 Russia regions bordering China, Mongolia, and Kazakhstan during summer 2024 (Table S2, Figure 1). These ticks belonged to the following 6 species: *Haemaphysalis concinna* (506 ticks), *Dermacentor reticulatus* (249 ticks), *Dermacentor silvarum* (122 ticks), *Dermacentor nuttalli* (39 ticks), *Ixodes persulcatus* (2349 ticks), and *Ixodes pavlovskyi* (179 ticks; Table 2).

### 3.2. qPCR Detection of SGLV in Ticks

Screening of 506 *Haemaphysalis concinna* tick samples for SGLV RNA yielded 11 positive specimens. qPCR Ct values ranged from 19.1 to 31.0. The overall prevalence of SGLV RNA in the studied *Haemaphysalis concinna* sample was 2.2% (11/506; 95% CI: 1.2–3.8). SGLV RNA-positive ticks were collected in the Republic of Altai, Amur Oblast, Jewish Autonomous Oblast, and Khabarovsk Krai (Table 3). The highest positivity rate was recorded in Amur Oblast, which borders China, at 5.8% (95% CI: 2.8–11.4). Of particular interest is the detection of an SGLV isolate in a tick from the Republic of Altai, representing the westernmost record of this virus to date. No SGLV RNA was detected in the tested ticks of the genera *Ixodes* and *Dermacentor*.

### 3.3. SGLV Genome Sequences

Full-length nucleoprotein-coding sequences of the S segment were determined for the detected SGLV isolates and deposited in the GenBank database under accession numbers PX461779-PX461789. The obtained sequences comprised 1524 nucleotides.

**Figure 2:**
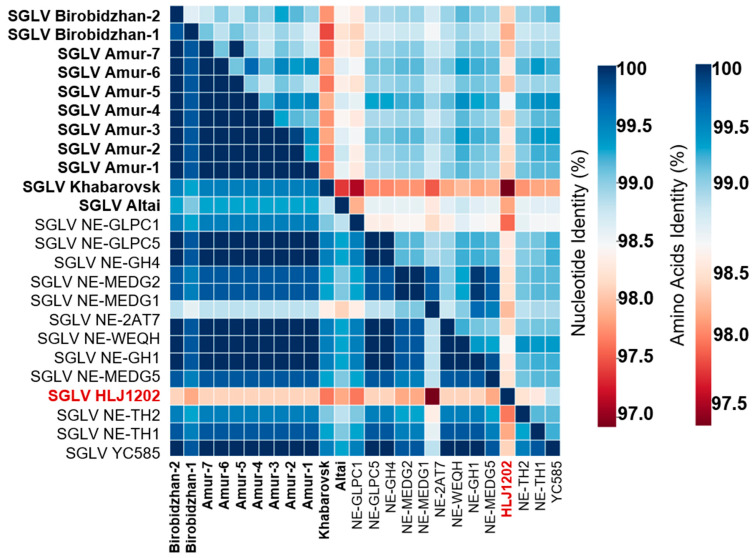
Pairwise nucleotide (upper triangle) and amino acid (lower triangle) sequence identities of SGLV isolates. Russian SGLV isolates identified in this study are highlighted in bold black. Isolates detected in *Homo sapiens* are highlighted in bold red. Other isolates from *Haemaphysalis* ticks are highlighted in black.

Nucleotide divergence among the Russian SGLV isolates ranged up to 1.2%, while the corresponding amino acid sequences showed ≤0.2% variation (Table S3). Nine of the 11 identified isolates were identical at the amino acid level. The SGLV Birobidzhan-1 isolate (GenBank ID: PX461788) carried a single-amino acid substitution, R177K, distinguishing it from the consensus sequence, whereas the SGLV Altai isolate (GenBank ID: PX461779) harbored two substitutions: V401A and E403D (Figure 2).

Subsequently, we compared the SGLV S segment sequences detected in this study with all available counterparts in the GenBank database. Of the 15 SGLV isolate sequences currently available in GenBank, two isolates, HLJ1202 (GenBank ID: NC_079002) and JLYB-2024-3 (GenBank ID: PV034577), were recovered from humans in China, while the remaining isolates were obtained from *Haemaphysalis concinna* ticks, also in China. The SGLV isolates recovered from humans exhibited the highest level of amino acid divergence compared to the Russian SGLV isolates, with 8 amino acid substitutions compared to the SGLV HLJ1202 isolate and 3 substitutions compared to the SGLV JLYB-2024-3 isolate. Among the SGLV isolates obtained from *Haemaphysalis* ticks, the SGLV NE-MEDG1 isolate (GenBank ID: PQ475701) proved to be the most divergent, demonstrating 5 amino acid substitutions. In contrast, the remaining SGLV isolates from *Haemaphysalis* ticks exhibited either 1-2-amino acid substitutions or were completely identical to the Russian SGLV isolates (Figure 2 and Table S3). The elevated amino acid divergence observed in human-derived isolates, despite the conserved nature of the nucleoprotein, may reflect lineage-specific variation.

### 3.4. SGLV Phylogeny

**Figure 3:**
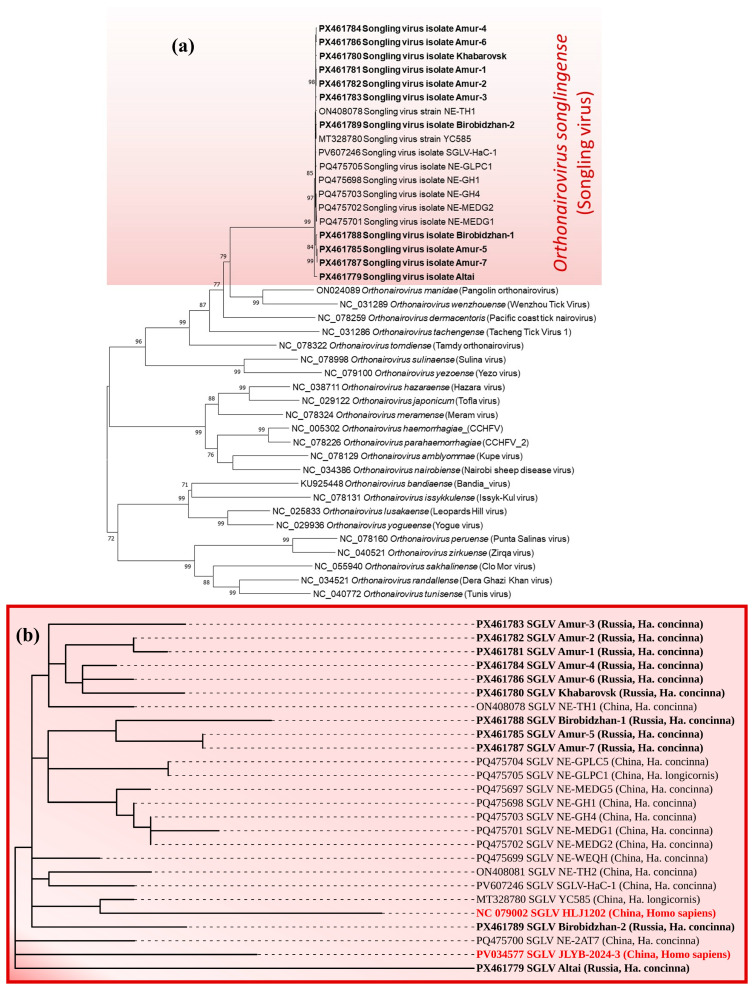
Phylogenetic relationships of SGLV and other *Orthonairovirus* species’ S segment sequences. (**a**) Phylogenetic tree constructed from complete coding S segment nucleotide sequences of SGLV and related orthonairoviruses. (**b**) Maximum-likelihood tree based on complete coding nucleotide sequences of the S segment, including Russian SGLV isolates identified in this study and reference SGLV sequences from GenBank. Viruses are indicated by GenBank accession number, name and isolate identifier, location of isolation, and host. Russian SGLV isolates identified in this study are highlighted in bold black. Isolates detected in *Homo sapiens* are highlighted in bold red. Other isolates from *Haemaphysalis* ticks are highlighted in black.

The phylogenetic analysis confirms that the Russian SGLV variants identified in this study belong to the *Orthonairovirus songlingense* species, forming a distinct cluster well-separated from other members of the *Orthonairovirus* genus. The clade formed by the SGLV isolates was most closely related to the clade comprising Pangolin orthonairovirus (*Orthonairovirus manidae*) and Wenzhou tick virus (*Orthonairovirus wenzhouense*), reflecting their putative phylogenetic affinity (Figure 3a). Phylogenetic analysis revealed no distinct clustering of known SGLV isolates based on either geographic origin or host source (*Haemaphysalis* ticks or *Homo Sapiens*). All known reference isolates originated from four provinces in Northeast China (Inner Mongolia, Heilongjiang, Yichun, and Jilin), which border the three studied Russian regions (Amur Oblast, Jewish Autonomous Oblast, and Khabarovsk Krai) (Figure 3).

Analysis of the S segment nucleotide sequences of the Russian SGLV isolates revealed that the Amur-5 (GenBank ID: PX461785), Amur-7 (GenBank ID: PX461787), and Birobidzhan-1 (GenBank ID: PX461788) isolates form a distinct monophyletic clade, clustering with reference variants detected in *Haemaphysalis* ticks from the Chinese provinces of Inner Mongolia, Heilongjiang, and Jilin. In contrast, the majority of detected SGLV isolates from Amur Oblast (Amur-1, Amur-2, Amur-3, Amur-4, Amur-6) and the isolate from Khabarovsk Krai (GenBank ID: PX461780) grouped into a separate clade together with the SGLV NE-TH1 isolate (GenBank ID: ON408078) from Heilongjiang Province, which was obtained from a *Haemaphysalis concinna* tick. The SGLV Birobidzhan-2 (GenBank ID: PX461789) and SGLV Altai (GenBank ID: PX461779) isolates were the most genetically divergent. The distant phylogenetic position of the SGLV Altai isolate was expected, as the Republic of Altai is geographically the most remote from the detection sites of all known SGLV isolates. Notably, however, the isolate from the Republic of Altai showed the closest genetic relationship to the SGLV JLYB-2024-3 isolate, which was recovered from a patient in 2024 (Figure 3b). The unexpected genetic proximity of the SGLV Altai isolate to a human-derived SGLV variant warrants further surveillance to determine whether this reflects transmission chains or convergent evolution under selective pressures.

### 3.5. SGLV Co-Infections

Natural foci of many tick-borne infections often overlap due to shared vectors and reservoir hosts, increasing the risk of co-infections. In this study, genetic material of both SGLV and *Rickettsia heilongjiangensis* was simultaneously detected in three *Haemaphysalis concinna* ticks from Amur Oblast and one *Haemaphysalis concinna* tick from the Jewish Autonomous Oblast (GenBank IDs: PX935561 and PX935562). The co-detection of SGLV and *Rickettsia heilongjiangensis* in individual ticks highlights the potential for simultaneous transmission of multiple pathogens to vertebrate hosts, warranting further investigation into clinical outcomes of such co-infections. Genetic markers of other tick-borne pathogens widely circulating in Russia, such as tick-borne encephalitis virus, *Borrelia burgdorferi* s.l., *Borrelia miyamotoi*, and *Anaplasma phagocytophilum*, were not detected in the tested *Haemaphysalis concinna* ticks. This finding is consistent with current knowledge, as *Haemaphysalis concinna* is not considered a primary vector for these agents.

Our study demonstrates that genetic markers of rickettsiae are relatively common in *Haemaphysalis concinna* ticks. The positivity rates in *Haemaphysalis concinna* were as follows: 35.1% for Jewish Autonomous Oblast (74/211; 95% CI: 28.9–41.7); 32.2% for Amur Oblast (39/121; 95% CI: 24.5–40.1); 21.9% for Khabarovsk Krai (23/105; 95% CI: 15.1–30.7); and 4.3% for Altai Republic (3/69; 95% CI: 1.5–12.1). For all rickettsia-positive samples, a fragment of the gltA gene (750 bp) was sequenced. Genotyping revealed that the majority of detected variants belonged to *Rickettsia heilongjiangensis*. However, *Candidatus Rickettsia rara* was identified in two ticks from Amur Oblast and one tick from the Jewish Autonomous Oblast. *Candidatus Rickettsia rara* has been previously reported in *Haemaphysalis concinna* and *Haemaphysalis japonensis* ticks collected in Amur Oblast and Khabarovsk Krai. The positivity rate of *Haemaphysalis concinna* with rickettsiae in Amur Oblast reported in the literature is 1.5%, which aligns well with our findings [23]. The identified *Rickettsia heilongjiangensis* variants were identical over the analyzed fragment to numerous reference isolates from China (054, GenBank ID: CP002912; NM-HLBE-35, GenBank ID: PX464593) and Russia (Choya-2017, GenBank ID: MN537887), but also showed 100% identity to several prototype variants of *Rickettsia japonica*. The detected *Candidatus Rickettsia rara* variants were identical over the gltA fragment to known isolates from Khabarovsk Krai and China (Kh-607Hc, GenBank ID: PP824725; NM-HLBE-261, GenBank ID: PX464606; FE1, GenBank ID: DQ365805). Multi-locus sequencing approaches may be required for reliable species-level identification.

## 4. Discussion

The present study provides the first molecular evidence of SGLV circulation in ticks collected in Russia. Prior to this work, SGLV had been reported in China, primarily in the northeastern provinces bordering Russia. Our findings extend the known geographic range of SGLV westward into Asian Russia, including the Republic of Altai, which represents the westernmost record of this virus to date. The detection of SGLV RNA in *Haemaphysalis concinna* ticks across four geographically distinct Russian regions suggests that the virus is not an incidental introduction but rather may be established in natural foci within Russian territory. This expansion is consistent with the known distribution of *Haemaphysalis concinna*, which ranges from Western Europe across Siberia to the Russian Far East and China, highlighting the potential for further westward spread of SGLV.

The exclusive detection of SGLV in *Haemaphysalis concinna* with no positive findings in *Ixodes* spp. and *Dermacentor* spp. supports a degree of vector specificity. Although SGLV has also been detected in *Haemaphysalis longicornis*, *Haemaphysalis flava*, and *Ixodes persulcatus* in China [13], our data suggest that *Haemaphysalis concinna* may serve as the primary or most efficient vector in the ecosystems surveyed. The absence of SGLV RNA in *Ixodes persulcatus* is noteworthy given that it is the predominant tick species in many parts of Asian Russia. This finding may reflect ecological segregation or differences in vector competence, which warrant further experimental investigation.

The overall positivity rate of *Haemaphysalis concinna* for SGLV RNA was 2.2% (95% CI: 1.2–3.8), which is comparable to or slightly lower than rates reported in China. The highest positivity rate was observed in Amur Oblast (5.8%, 95% CI: 2.8–11.4). This region directly borders China’s Heilongjiang Province. Notably, multiple SGLV isolates have already been obtained from both ticks and humans in that Chinese province. This geographic proximity likely facilitates virus exchange across the border through tick dispersal or host movement. Conversely, SGLV RNA was detected in the Republic of Altai, the most geographically distant surveyed region. Despite having the shortest border with China, the presence of SGLV in this area suggests either an independent enzootic focus or long-distance dispersal via migratory birds or large mammals. These mechanisms are well-documented for other tick-borne pathogens [24,25].

Phylogenetic analysis of the S segment nucleoprotein-coding sequences revealed that Russian SGLV isolates form a clade within *Orthonairovirus songlingense*. Notably, the majority of isolates from Amur Oblast and Khabarovsk Krai clustered with the Chinese SGLV NE-TH1 isolate (GenBank ID: ON408078) from Heilongjiang Province. In contrast, the SGLV Altai isolate (GenBank ID: PX461779) was the most genetically divergent among Russian isolates and showed unexpected phylogenetic proximity to the human-derived SGLV JLYB-2024-3 isolate (GenBank ID: PV034577) from Jilin Province. This finding is striking given the considerable geographic distance between the Altai Republic and Jilin Province (~3500 km).

The amino acid divergence observed between human-derived SGLV isolates and Russian tick-derived SGLV isolates deserves special attention. Despite the conserved nature of the nucleoprotein, human-derived isolates carried 3-8-amino acid substitutions compared to the Russian SGLV isolates. Whether these substitutions represent host-specific adaptations or are merely markers of distinct viral lineages requires functional investigation using reverse genetics systems. Previous studies have demonstrated that SGLV isolates from patients induce pronounced cytopathic effects in human hepatocellular carcinoma cells, under-scoring the virus’s pathogenic potential [13]. The detection of SGLV in the Republic of Altai is particularly concerning as the isolate shares genetic similarity with a human-derived Chinese variant. This finding raises concerns about the possibility of undiagnosed SGLV infections in Russia. Such cases may easily go unnoticed as SGLV infection presents with nonspecific clinical symptoms.

An important finding of this study is the co-detection of SGLV and *Rickettsia heilongjiangensis* in four individual *Haemaphysalis concinna* ticks. *Rickettsia heilongjiangensis* is a recognized pathogen causing Far Eastern spotted fever, and its co-circulation with SGLV in the same tick species and geographic regions raises the possibility of co-transmission to humans. Mixed infections with multiple tick-borne pathogens have been associated with more severe clinical outcomes, although the pathophysiological interactions between orthonairoviruses and rickettsiae remain unexplored. The relatively high positivity rates of *Haemaphysalis concinna* with *Rickettsia heilongjiangensis* underscore the need for diagnostic panels capable of detecting both viral and bacterial tick-borne agents in patients presenting with febrile illness after tick bites.

Several limitations of this study should be acknowledged. First, we focused on the S segment for molecular detection and phylogenetic analysis. While this conserved region is suitable for broad surveillance and taxonomic classification, sequencing of the M and L segments would provide higher resolution for phylogeographic studies and could reveal reassortment events, which are known to occur among orthonairoviruses. Therefore, future studies of SGLV complete genome sequencing are essential. Second, we did not attempt virus isolation from positive tick pools, which would enable cytopathic effect assessment in cell lines. Third, the absence of SGLV detection in *Ixodes* spp. and *Dermacentor* spp. ticks does not definitively exclude their potential as vectors as sample sizes for some species were limited. Finally, serological surveys of human populations and vertebrate reservoirs in the surveyed regions are needed to assess the zoonotic risk.

Despite these limitations, our study has important public health and clinical implications. The demonstration of SGLV circulation in four Russian regions, including areas with high tourism and agricultural activity, mandates the inclusion of SGLV in the differential diagnosis of tick-borne febrile illnesses in Russia.

## 5. Conclusions

This study establishes the presence of SGLV in *Haemaphysalis concinna* ticks across a wide geographic range in Asian Russia, from the Altai Republic in the west to Khabarovsk Krai in the east. Proactive surveillance of emerging tick-borne orthonairoviruses remains a critical priority for public health preparedness.

## Figures and Tables

**Table 1:** Oligonucleotide primers used for targeted library enrichment for NGS.

Oligonucleotide Name	Oligonucleotide Sequence	Coordinate	Amplification Fragment Size	Annealing Temperature
SGLV_S_F1	CCTGTCAACCCTCACCTACTC	221	333	62 °C
SGLV_S_R1	CTCATACTTTATCGCATTCCTCCA	554		60 °C
SGLV_S_F2	CCTGTCAACCCTCACCTACTC	221	696	62 °C
SGLV_S_R2	CTCTCCCATTGCCTCTACCT	917		60 °C
SGLV_S_F3	GGACAAAGAAGAACAAAGGAGG	781	656	59 °C
SGLV_S_R3	GAAACAGGGCACAGATTGAC	1437		59 °C
SGLV_S_F4	TGGAGGAATGCGATAAAGTATGAG	531	906	60 °C
SGLV_S_R4	GAAACAGGGCACAGATTGAC	1437		59 °C
SGLV_S_F5	ACAGTTGACGGGTTCCTCTC	981	671	61 °C
SGLV_S_R5	CTGGCTGTGGTCTCTGAGTG	1652		62 °C

**Table 2:** Information about tick sample collection in Asian Russia.

Russian Region	*Haemaphysalis concinna*	*Dermacentor reticulatus*	*Dermacentor silvarum*	*Dermacentor nuttalli*	*Ixodes* *persulcatus*	*Ixodes pavlovsky*	Total
Altai Republic	69	19	24	39	79	-	230
Amur Oblast	121	-	-	-	648	-	769
Jewish Autonomous Oblast	211	-	-	-	81	-	292
Khabarovsk Krai	105	-	98	-	59	-	262
Primorsky Krai	-	-	-	-	434	-	434
Tomsk Oblast	-	230	-	-	138	179	547
Krasnoyarsk Krai	-	-	-	-	200	-	200
Republic of Tyva	-	-	-	-	210	-	210
Republic of Khakassia	-	-	-	-	150	-	150
Irkutsk Oblast	-	-	-	-	200	-	200
Zabaykalsky Krai	-	-	-	-	150	-	150
Total:	506	249	122	39	2349	179	3444

**Table 3:** Prevalence of SGLV in *Haemaphysalis concinna* ticks in Asian Russia.

Russian Region	No. Positive Ticks/ No. Ticks Examined	Prevalence, % (95% CI)	SGLV Isolate Name (GenBank ID)	Geographic Location Coordinates	qPCR Ct
Altai Republic	1/69	1.4 (0.3–7.7)	Altai (PX461779)	51.996694, 86.478250	27.5
Khabarovsk Krai	1/105	0.9 (0.2–5.2)	Khabarovsk (PX461780)	46.744194, 134.229889	26.1
Amur Oblast	7/121	5.8 (2.8–11.4)	Amur-1 (PX461781)	53.400431, 124.080654	19.1
			Amur-2 (PX461782)	53.400431, 124.080654	23.5
			Amur-3 (PX461783)	52.101272, 127.954997	21.7
			Amur-4 (PX461784)	51.510731, 128.356803	29.3
			Amur-5 (PX461785)	49.745496, 129.841354	27.2
			Amur-6 (PX461786)	49.458277, 130.093075	31.0
			Amur-7 (PX461787)	49.449532, 130.185823	27.7
Jewish Autonomous Oblast	2/211	0.9 (0.3–3.4)	Birobidzhan-1 (PX461788)	48.939917, 132.661889	23.4
			Birobidzhan-2 (PX461789)	48.939917, 132.661889	28.7
Total:	11/506	2.2 (1.2–3.8)			

## Data Availability

The original contributions presented in the study have been deposited in GenBank under accession numbers PX461779-PX461789 for SGLV and PX935561-PX935562 for *Rickettsia*, and are included in the article/Supplementary Materials. Further inquiries can be directed to the corresponding author.

## Supplementary Materials

The following supporting information can be downloaded at: https://www.mdpi.com/article/10.3390/v18060688/s1, Table S1: Viral sequences using for analysis in this study; Table S2: Geographic coordinates of tick collection; Table S3: Identity level of nucleotide and amino acid sequences (%) of identified Russian SGLV isolates compared to SGLV prototype isolates.

## Abbreviations

The following abbreviations are used in this manuscript:

SGLV	Songling virus
CCHFV	Crimean-Congo hemorrhagic fever virus
ssRNA(-)	single-stranded negative-sense RNA
RNP	ribonucleoprotein
qPCR	quantitative PCR
cDNA	complementary DNA
dsDNA	double-stranded DNA
95% CI	95% confidence interval
