# Supplementary material for: First Report of Orthonairovirus songlingense in Haemaphysalis concinna Ticks from Russia
Source: Viruses. 2026 Jun 22;18(6):688. doi: 10.3390/v18060688 (PMC13307660; doi:10.3390/v18060688)
Supplement: Supplementary file 1 [file viruses-18-00688-s001.zip › Table S1 and Table S2.pdf]

Table S1. Viral sequences using for analysis in this study.

No.	GenBank ID	Virus name	Isolate/strain	Taxonomy	Isolation source	Geographic location name	Collection data
1	PV034577	Songling virus	JLYB-2024-3	*Orthonairovirus songlingense*	*Homo sapiens*	China: Yanji	2024
2	PQ475705	Songling virus	NE-GLPC1	*Orthonairovirus songlingense*	*Haemaphysalis longicornis*	China: Helong, Jilin	2023
3	PQ475704	Songling virus	NE-GPLC5	*Orthonairovirus songlingense*	*Haemaphysalis concinna*	China: Helong, Jilin	2023
4	PQ475703	Songling virus	NE-GH4	*Orthonairovirus songlingense*	*Haemaphysalis concinna*	China: Genhe, Inner Mongolia	2022
5	PQ475702	Songling virus	NE-MEDG2	*Orthonairovirus songlingense*	*Haemaphysalis concinna*	China: Ergun, Inner Mongolia	2022
6	PQ475701	Songling virus	NE-MEDG1	*Orthonairovirus songlingense*	*Haemaphysalis concinna*	China: Ergun, Inner Mongolia	2022
7	PQ475700	Songling virus	NE-2AT7	*Orthonairovirus songlingense*	*Haemaphysalis concinna*	China: Hunchun, Jilin	2023
8	PQ475699	Songling virus	NE-WEQH	*Orthonairovirus songlingense*	*Haemaphysalis concinna*	China: Yakeshi, Inner Mongolia	2022
9	PQ475698	Songling virus	NE-GH1	*Orthonairovirus songlingense*	*Haemaphysalis concinna*	China: Genhe, Inner Mongolia	2022
10	PQ475697	Songling virus	NE-MEDG5	*Orthonairovirus songlingense*	*Haemaphysalis concinna*	China: Tahe, Heilongjiang	2022
11	NC 079002	Songling virus	HLJ1202	*Orthonairovirus songlingense*	*Homo sapiens*	China: Heilongjiang, Lanxi	2002
12	ON408081	Songling virus	NE-TH2	*Orthonairovirus songlingense*	*Haemaphysalis conicinna*	China: Tahe, Heilongjia	2021

						ng	
13	ON408078	Songling virus	NE-TH1	*Orthonairovirus songlingense*	*Haemaphysalis conicinna*	China: Tahe, Heilongjiang	2021
14	MT328780	Songling virus	YC585	*Orthonairovirus songlingense*	*Haemaphysalis longicornis*	China: Heilongjiang, Yichun	2005
15	PV607246	Songling virus	SGLV-HaC-1	*Orthonairovirus songlingense*	*Haemaphysalis concinna*	China: Hulunbeier, Inner Mongolia	2024
16	MT328777	Songling virus	HLJ1202	*Orthonairovirus songlingense*	*Homo sapiens*	China: Heilongjiang, Lanxi	2002

Table S2. Geographic coordinates of tick collection.

Russia region	No.	Latitude (°)	Longitude (°)
Altai Republic	1	50.358772	87.055275
	2	50.383078	87.019332
	3	51.9758259	85.8660439
	4	51.9818072	85.9077997
	5	51.9542866	85.8922626
	6	51.9012919	85.9145971
	7	51.9716385	86.019472
	8	51.956923	86.332688
	9	51.688805	86.339569
	10	51.5816	86.302077
	11	51.996681	86.478257
	1	49.468778	130.1587
	2	49.661265	129.761414

Amur (Amur Oblast)	3	49.83266	129.883229
	4	51.653792	128.175201
	5	52.123887	128.209606
	6	52.027634	127.685832
	7	51.842521	127.136516
	8	53.488253	123.896012
	9	53.731529	123.852688
	10	49.449532	130.185823
	11	49.458277	130.093075
	12	49.745496	129.841354
	13	51.510731	128.356803
	14	52.101272	127.954997
	15	53.400431	124.080654
Irkutsk (Irkutsk Oblast)	1	52.0861776	104.5878482
	2	52.0823634	104.5864047
	3	52.0712737	104.594777
	4	52.0752663	104.5846725
	5	52.0647073	104.6066136
	6	52.0739355	104.6183059
	7	52.073625	104.5934894
	8	52.0705822	104.5876204
	9	52.056276	104.6209042
	10	52.0518378	104.621626
	11	52.0321271	104.6454436
	12	52.0268872	104.6428453
	13	52.0579624	104.6646421

	14	52.026976	104.6639204
	15	52.0622224	104.6357722
	16	52.0283083	104.6478976
	17	52.0498849	104.6556924
	18	52.045091	104.643134
	19	52.0573411	104.6203268
	20	52.0244891	104.645588
	21	52.0139181	104.663776
	22	52.0115192	104.6766231
	23	52.0151618	104.6812423
Jewish Autonomous Oblast	1	48.698025	132.804158
	2	48.939902	132.661899
Khabarovsk (Khabarovsk Krai)	1	48.494603	135.14948
	2	48.460643	135.177693
	3	47.24863	134.39577
	4	47.24871	134.39581
	5	47.24884	134.39593
	6	46.744204	134.229876
	7	47.24863	134.39577
Krasnoyarsk (Krasnoyarsk Krai)	1	53.0823241	91.4874499
	2	53.0270209	91.5011828
	3	55.409938	89.08702
	4	55.411612	89.125355
	5	55.5483	89.184555
	6	55.46873	89.153362
Primorsky Krai	1	44.0831435	131.4233905

	2	44.1704174	131.6892353
	3	44.256722	131.6610744
	4	44.4289518	131.379466
	5	44.3297036	131.5396915
	6	44.5058746	131.5328941
	7	44.6386909	131.7678914
	8	44.7201672	131.6727272
	9	44.5702454	131.562026
	10	44.635236	131.5513443
	11	45.6819526	134.0987887
	12	45.5542634	134.1764737
	13	45.4984781	134.2434771
	14	45.6405534	133.8181513
	15	45.3422072	134.0441186
	16	45.4022391	134.0833817
	17	45.4821403	134.0434381
	18	45.5732994	134.2065767
	19	45.6446268	133.8239777
	20	45.7517867	134.1026729
	21	45.3728234	133.6980462
	22	45.5279307	133.7516046
	23	43.806448	131.676556
	24	43.940954	131.582421
Republic of Khakassia	1	52.9625929	90.7136106
	2	52.9314589	90.7455396
	3	52.925664	90.6624555

	4	52.8991732	90.7408499
	5	52.8927855	90.6816187
	6	52.8776624	90.8694163
	7	53.0567449	91.4091723
	8	53.0592209	91.2773364
	9	53.0171084	91.4599841
	10	53.0765494	91.3707202
Republic of Tyva	1	51.5934947	95.3520149
	2	51.5738683	95.3654045
	3	51.5702407	95.3657478
	4	51.5644787	95.3650612
	5	52.4354073	96.4884974
	6	52.429424	96.5176241
	7	52.4358961	96.5172158
	8	52.4212081	96.5372216
	9	52.4326602	96.5396713
	10	52.4398785	96.5376299
	11	52.4470956	96.5070088
	12	52.4423672	96.4539321
	13	52.433158	96.420453
	14	52.4256897	96.4229027
	15	52.4357081	96.4813861
	16	52.4318358	96.5114108
	17	52.4364121	96.4120983
	18	52.4262028	96.4172949
	19	52.3920371	96.3607099

	20	52.3811126	96.3762997
	21	52.433596	96.507369
	22	52.4184562	96.4490518
	23	52.4092994	96.4554032
	24	52.4240902	96.304125
	25	52.4325399	96.2792969
	26	52.4202169	96.2654393
	27	51.0493628	94.5409741
	28	51.0484805	94.5442404
	29	51.0450635	94.5352582
	30	51.0430194	94.5328877
	31	51.0406153	94.5380465
	32	51.0380203	94.5305511
Tomsk (Tomsk Oblast)	1	56.446808	84.97754
	2	56.45082	84.980653
	3	56.443435	85.005635
	4	56.447852	84.968828
	5	56.452972	85.00157
	6	56.449915	84.957112
	7	56.468051	85.033955
	8	56.406504	84.989166
	9	56.400464	84.984288
	10	56.47567	85.041239
	11	56.375149	85.100275
	12	56.359071	85.350902
	13	56.51537	85.05617

	14	56.480735	84.872351
	15	56.439979	85.040628
	16	56.387588	85.162191
	17	56.34296	85.364326
	18	56.220939	84.842221
	19	56.382609	84.099596
	20	56.565771	84.044919
Zabaykalsk (Zabaykalsky Krai)	1	50.8963443	113.2271171
	2	50.9086954	113.2262699
	3	50.9242852	113.2820464
	4	50.9031551	113.1867298
	5	50.9079041	113.1700312
	6	50.8831394	113.2886865
	7	50.9246492	113.2245953
	8	50.9046903	113.2496545
	9	50.8852184	113.2216624
	10	50.8910463	113.250821
	11	50.8990585	113.1962569
